# Surface-associated MUC5B mucins promote protease activity in *Lactobacillus fermentum* biofilms

**DOI:** 10.1186/1472-6831-13-43

**Published:** 2013-09-08

**Authors:** Claes Wickström, Luis Chávez de Paz, Julia R Davies, Gunnel Svensäter

**Affiliations:** 1 Department of Oral Biology, Faculty of Odontology, Malmö University, Malmö 20506, SE, Sweden

**Keywords:** Lactobacilli, Proteolytic activity, Proteolysis, Mucus glycoprotein

## Abstract

**Background:**

Mucosal surfaces are coated with layers of mucus gel that protect the underlying tissues and promote colonization by members of the commensal microflora. *Lactobacillus fermentum* is a common inhabitant of the oral cavity, gastrointestinal and reproductive tracts and is one of the most important lactic acid bacteria contributing to the formation of a healthy intestinal microflora. We have investigated the proteolytic activity in *L. fermentum* in response to interactions with the MUC5B mucin, which is a major component of mucus gels at sites colonized by this micro-organism.

**Methods:**

Biofilms of *Lactobacillus fermentum* were established in mini-flow cells in the presence or absence of human salivary MUC5B. The proteolytic activity of biofilm cells was examined in a confocal scanning laser microscope with a fluorescent protease substrate. Degradation of MUC5B by *L. fermentum* was analysed using SDS-PAGE followed by Western blotting with antisera raised against the MUC5B peptide. Cell surface proteins differentialy expressed in a MUC5B-rich environment were identified with the aid of comparative two-dimensional electrophoresis followed by LC-MS/MS.

**Results:**

*Lactobacillus fermentum* adhered well to surfaces coated with MUC5B mucin and in biofilms of *L. fermentum* formed in a MUC5B environment, the proportion of proteolytically-active cells (47 ± 0.6% of the population), as shown by cleavage of a fluorescent casein substrate, was significantly greater (p < 0.01) than that in biofilms formed in nutrient broth (0.4 ± 0.04% of the population). Thus, the presence of MUC5B mucins enhanced bacterial protease activity. This effect was mainly attributable to contact with surface-associated mucins rather than those present in the fluid phase. Biofilms of *L. fermentum* were capable of degrading MUC5B mucins suggesting that this complex glycoprotein can be exploited as a nutrient source by the bacteria.

Comparison of the surface proteomes of biofilm cells of *L. fermentum* in a MUC5B environment with those in nutrient broth using two-dimensional electrophoresis and mass spectroscopy, showed that the enhanced proteolytic activity was associated with increased expression of a glycoprotease; *O-*sialoglycoprotein endopeptidase, as well as chaperone proteins such as DnaK and trigger factor.

**Conclusions:**

Adhesion to mucin-coated surfaces leads to a shift towards a more protease-active phenotype within *L. fermentum* biofilms and proteases produced within the biofilms can degrade MUC5B mucins. The enhanced proteolytic activity was associated with an increase in *O-*sialoglycoprotein endopeptidase on the cell surface. We propose that the upregulation of chaperone proteins in the mucin environment may contribute to the protease-active phenotype through activation of the glycopeptidase. This would represent one way for commensal lactobacilli *e.g. L. fermentum* to exploit complex substrates in their local environment in order to survive on mucosal surfaces.

## Background

The body’s mucosal surfaces are colonized by a large number of commensal bacterial species which co-exist in a mutualistic relationship with their human host. These microbial communities, or biofilms, are believed to constitute the first line of defense against infection by competitively inhibiting non-indigenous organisms that may cause disease. In the gut, the commensal flora has been proposed to have an important influence on development, immunity and nutrition (for reviews see [1-3]). Despite their importance, surprisingly little is known about how the members of these communities survive in the mucosal environment and interact with each other, and the host, to form a dynamic ecosystem. *Lactobacillus* represents the most numerous and diverse group among lactic acid bacteria that inhabit mucosal surfaces in humans, including the gastrointestinal tract [4], female reproductive tract [5] and the oral cavity [6]. Lactobacilli are generally viewed as conferring beneficial biological effects to the host. For example, in the gastrointestinal tract, lactobacilli promote immune stimulation and reinforcement of mucosal defence [7]. Amongst the lactobacilli, *Lactobacillus fermentum* is a common inhabitant of the gastrointestinal tract [8], including the oral cavity [9,10]. In contrast to the beneficial role in the intestine, Lactobacilli in the oral cavity are often associated with carious disease [6] and *Lactobacillus fermentum* is frequently isolated from dentine caries lesions in children, implying a role in the caries process [11].

Mucosal surfaces are protected by a layer of mucus gel derived from mucin-producing cells in the underlying epithelia. Mucus gels are composed of large, polymeric gel-forming glycoproteins belonging to the mucin protein family and the mucin species comprising these gels on different mucosal surfaces may vary. For instance, MUC5B is a predominant mucin in the oral cavity, female reproductive tract and airways [12] while MUC5AC, MUC6 and MUC2 are found at different sites throughout the gastrointestinal tract [13]. The large polymeric mucins are composed of subunits linked by disulphide bonds, and within each subunit stretches of naked protein backbone alternate with highly glycosylated regions containing large numbers of oligosaccharide side chains [14]. Lactobacilli bind to both gastric and intestinal mucins [15] and a mucin-binding protein (32-Mmubp), which is a component of the ABC transporter system, has been identified in *L. fermentum*[16]. Interactions with mucins most likely allow lactobacilli to be retained within the mucus layer where they contribute to the formation of multi-species biofilms.

Currently, the mechanisms regulating survival and growth of lactobacilli on mucosal surfaces are largely unresolved. However, since lactic acid bacteria are only able to take up short peptides via transport systems, that are specific for oligopeptides (Opp) and di-tripeptides (DtpT) [17], growth and survival in biofilms in mucus environments will most likely depend on the ability of the bacteria to degrade complex substrates such as mucins. The ability of bacteria to utilize mucins as their sole nutrient source has been demonstrated for *Akkermansia muciniphilia* which was isolated from a faecal sample from a healthy adult [18]. Bioinformatics studies reveal that the genome of *L. fermentum* strain 28-3-CHN encodes at least 10 proteases. Of these, several are predicted to be extracellular and therefore have the potential to play a role in the generation of nutrients from mucins on mucosal surfaces. In the present study we investigate how proteolytic activity in biofilms of *L. fermentum* is related to the environment surrounding the bacteria; a mucin-rich environment or protein-rich nutrient broth medium, as well as whether large gel-forming mucins can be degraded by *L. fermentum* as a potential source of nutrients. In addition, changes in cell-surface protein expression associated with enhancement of proteolytic activity were examined.

## Methods

### Bacterial strain

In order to investigate the naturally occurring interactions between MUC5B and *Lactobacillus fermentum,* a clinical strain known to be able to survive and grow in a mucus-rich environment was isolated from dental plaque. The strain, which originated from approximal supra-gingival plaque from a healthy 30-year old male*,* was identified by selective growth in Rogosa medium and fermentation tests using the API-50 CHL system (BioMerieux, Marcy l’Etoile, France). Identification as *L. fermentum* was confirmed by sequencing of the *pheS* gene [19]. Bacteria were stored at −80°Cand sub-cultured twice on blood agar in an atmosphere of 5% CO_2_ in air at 37°C for 48 hours before use.

### Isolation of human salivary MUC5B

Human salivary mucin MUC5B was isolated as previously described [20]. Whole saliva was collected on ice from eight volunteers, pooled and then mixed with an equal volume of 0.2 M NaCl and stirred overnight at 4°C. After gentle centrifugation (4400 g for 30 min at 4°C), the supernatant was subjected to isopycnic density-gradient centrifugation in CsCl/0.1 M NaCl (Beckman Optima LE-80 K, rotor 50.2Ti, 36,000 r.p.m., 96 h, 15°C, start density 1.45 g/ml; Beckman, Fullerton, CA, USA) and 22 fractions were collected. The MUC5B-containing fractions were pooled, dialysed against PBS (0.15 M NaCl, 5 mM NaH_2_PO_4_, pH 7) and stored at −20°C until use. To determine the concentration of MUC5B, aliquots of the MUC5B-containing fractions were pooled, dialyzed against water, lyophilized, and weighed (0.3 mg ml^-1^). The pool of MUC5B was subjected to sodium dodecyl sulfate–poly-acrylamide gel electrophoresis (SDS–PAGE), to examine whether low-molecular-weight proteins associated with the gel network contaminated the preparation.

### Biofilm formation

*L. fermentum* colonies from blood agar were suspended in nutrient broth [Todd-Hewitt broth (Difco Laboratories, Detroit, MI, USA)] or in a MUC5B solution in PBS to give OD_600_ values of 0.4 ± 0.01 (corresponding to approximately 1×10^6^ cells ml^–1^). Biofilms were established in mini-flow cell Ibidi μ-slides (Integrated Bio Diagnostics, Martinsried, Germany) by inoculating 120 μl of the suspension into the channels and incubating in humidified 5% CO_2_ in air at 37°C for 24 hours. The channels were then rinsed with PBS to remove non-adherent cells. In some cases, the slides were pre-conditioned with 100 μl of MUC5B + 10 μl 10 mM CaCl_2_ overnight in humidified 5% CO_2_ in air at 37°C to obtain a MUC5B coating. The slides were rinsed with PBS prior to biofilm formation. Biofilm formation was confirmed by staining with the BacLight™ LIVE/DEAD kit (Molecular Probes Inc., OR, USA) and visualization using a Nikon Eclipse TE2000 inverted confocal scanning laser microscope (CSLM).

### Proteolytic activity

To investigate the proteolytic activity of planktonic cells, *L. fermentum* cells were examined with the fluorescent protease assay kit QuantiCleave™ (Pierce, Rockford, IL, USA) as described previously [21]. Briefly, cells were incubated for 1 hour at 37°C with the FITC labeled casein substrate at a concentration of 10 μg ml^-1^ in 25 mM Tris with 150 mM NaCl and adjusted to pH 7.2. Aliquots were then introduced into mini-flow-cell Ibidi μ-slides and counterstained with the red fluorescent DNA-staining dye SYTO62® (Molecular Probes Inc., OR, USA) for 10 minutes at room temperature. For biofilm cells, the fluorescent substrate and counterstain were introduced directly into the mini-flow cells in which the biofilms were growing. Cells were then visualized using CSLM and 20 random images from each biofilm slide were captured using a motorized station connected to the Nikon software of the microscope. Each image contained more than 1000 bacterial cells and the proportion of red and green cells for each biofilm slide, corresponding to approximately 20,000 cells, was analysed using the software package *bio*Image_L [22]. Experiments were repeated three times and the mean percentage value for proteolytically active cells was calculated. Results were then further analyzed using the Mann–Whitney *U* test and p values of less than 5% were regarded as significant.

### Analysis of degradation of MUC5B by *L. fermentum* biofilms

Mini flow-chambers were pre-conditioned with MUC5B and *L. fermentum* cells then inoculated in nutrient broth. After 24 hours, the medium was removed and MUC5B mucins suspended in PBS were added to the biofilm for an additional 24 hours. After this time, the fluid in the flow-cell was collected with a pipette and centrifuged to remove bacterial cells (10000 g, 5 min, 4°C). The mucin-containing supernatant, as well as a control (MUC5B mucins incubated for 24 hours at 37°C but not exposed to *L. fermentum*) was analysed using SDS-PAGE under denaturing conditions followed by Western blotting. Samples (15 μl of the supernatant) were mixed with 5 μl NuPAGE LDS sample buffer (Invitrogen, Carlsbad, CA, USA) and run on NuPAGE 4–12% BisTris gels (Invitrogen, Carlsbad, CA, USA) at 180 V for 1 hour. The gels were electro-blotted onto PVDF membranes (Millipore, Immobilon-P, 0.45 mm, Bedford, MA, USA) overnight using a Mini Trans-Blot Electrophoretic Transfer Cell (Bio-Rad, Hercules, CA, USA) and the membranes then blocked with 5% (w/v) dry milk in TBST (20 mM Tris, 137 mM NaCl, 0.05% Tween 20) for 1 hour. MUC5B mucins were detected using an antiserum (LUM5B-14), raised against a synthetic peptide with the sequence CRAENYPEVSIDQVGQVL present in the third Cys domain of the MUC5B peptide, diluted 1:1000 in 5% BSA/TBST. The membranes were then incubated (1 hour) with a horseradish peroxidase-conjugated goat anti-rabbit antibody (Dako, P0448, Glostrup, Denmark) diluted 1:2500 in 5% skim milk/TBST, and bands were visualized using the ECL Western detection kit (Pierce, Rockford, IL, USA).

### 2DE, LC-MS/MS and Western blotting of cell surface proteins

The bulk phase surrounding biofilm cells growing in a MUC5B- or a nutrient broth- environment, was removed with a pipette from the flow-cells and replaced with 50 μl of extraction buffer consisting of 5 M urea, 2 M thiourea, 2% CHAPS, 2% caprylyl sulfobetaine, 2 mM tributyl phosphine, 0.5-2% IPG and 40 mM Tris base adjusted to pH 9.5. In order to solubilize surface proteins from the remaining biofilm cells, the flow-cells were incubated with shaking for 1 hour at room temperature and the extraction buffer then removed and centrifuged for 10 minutes (6000 *g*). The protein concentrations in the resulting supernatants were determined using a 2D Quant kit (GE Healthcare Life Sciences, Little Chalfont, UK). The supernatants were then diluted with rehydration buffer containing 8 M urea, 2% CHAPS, 10 mM DTT, 2% immobiline (IPG) buffer (GE Healthcare Life Sciences, Little Chalfont, UK) to give a final protein concentration of 20 μg in 200 μl. These samples were placed in re-swelling cassettes with 11 cm IPG strips, pH range 4–7, on top and rehydration was performed under oil at room temperature for 30 hours. Isoelectric focussing was carried out essentially as described by Davies *et al.*[23], using the Multiphor II (Amersham Pharmacia Biotech, Little Chalfont, UK), for 85, 500 volt hours. The strips were equilibrated and then embedded on top of 14% polyacrylamide gels. SDS-PAGE was performed at a constant current of 15 mA/gel, 10°C, over-night in a PROTEAN II xi cell (Bio-Rad, Hercules, CA) with high-range Rainbow molecular-weight standards (GE Healthcare Life Sciences, Little Chalfont, UK) on the acidic side of the IPG strips. Gels were stained with silver according to the manufacturer’s instructions or colloidal Coomassie Brilliant Blue G for protein identification. Gels were analyzed using Delta2D software (Decodon GmbH, Greifswald, Germany). After identification of the spot boundaries, the integrated optical density (IOD) values were determined and expressed as a percentage of the total intensity per gel. Spots showing more than a three-fold difference were cut from the Coomassie-stained gels, subjected to in-gel tryptic digestion and analysed by LC-MS/MS as described [23]. For detection of *O-*sialoglycoprotein endopeptidase, 2DE gels were electro-blotted onto PVDF membranes (Millipore, Immobilon-P, 0.45 mm, Bedford, MA, USA) overnight using a Mini Trans-Blot Electrophoretic Transfer Cell (Bio-Rad, Hercules, CA, USA) and the membranes then blocked with 5% (w/v) dry milk in TBST (20 mM Tris, 137 mM NaCl, 0.05% Tween 20) for 1 hour. The protein was detected using a polyclonal antiserum MOB-LFGE-2 raised against a synthetic peptide with the sequence (CDAAGEAYDKVGRV) (Innovagen AB, Lund, Sweden), diluted 1:1000 in 5% BSA/TBST. The membranes were then incubated (1 hour) with a horseradish peroxidase-conjugated goat anti-rabbit antibody and bands were visualized using the ECL Western detection kit as described above.

## Results

### Comparison of proteolytic activity in planktonic and biofilm cells of *L. fermentum*

**Figure 1:**
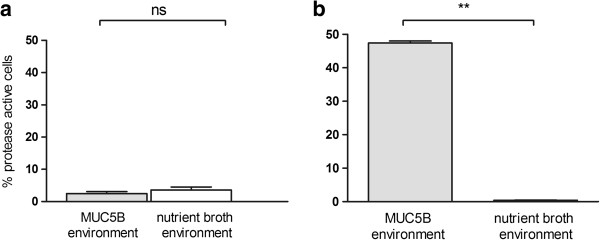
**Proportion of proteolytically-active *Lactobacillus fermentum *cells in planktonic and biofilm culture.** Graphs showing the percentage of proteolytically active cells in populations of **(a)** planktonic or **(b)** biofilm cells of *L. fermentum* in a MUC5B- or a nutrient broth-environment as revealed using a fluorescent substrate combined with CSLM. The bars represent the mean value of three independent experiments ± SE (** p < 0.01).

**Figure 2:**
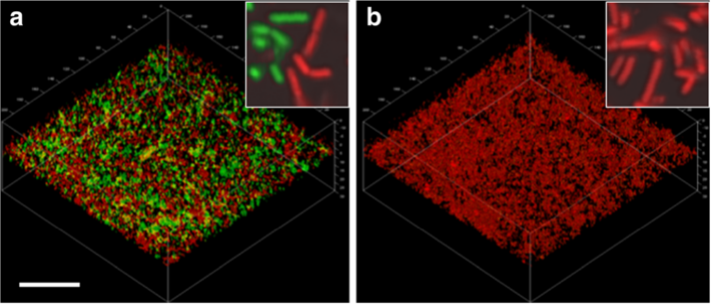
**Proteolytic activities of *Lactobacillus fermentum *biofilm populations in the presence or absence of MUC5B mucins.** CSLM images showing biofilm cells growing in **(a)** the presence of a MUC5B environment or **(b)** a nutrient broth environment. Proteolytic activity was visualized by incubation with a FITC-conjugated casein substrate (green) for 1 hour. Cells were then counterstained using Syto 24 (red). The bar represents 20 μm and the inserts show four-fold enlargements of the main micrographs.

To investigate the effect of MUC5B on proteolytic activity in planktonic cells of *L. fermentum*, cells were exposed to MUC5B in the fluid phase. Nutrient broth was used as a control. The proportion of proteolytically-active cells was evaluated using CLSM after incubation with FITC-casein. This revealed no difference between the proportion of proteolytically-active cells in nutrient broth to those in MUC5B mucins suspended in PBS, and in both cases the activity in the population was lower than 4% (Figure 1). No difference in the level of activity of the cell population suspended in MUC5B mucins in PBS and that suspended in PBS alone (control) was seen (data not shown). To compare the proteolytic activity of planktonic cells with that of biofilm cells of *L. fermentum,* bacteria were allowed to form biofilms under static conditions in a mini-flow chamber system for 24 hours in a MUC5B environment (on surfaces coated with MUC5B and MUC5B in the fluid phase) or a nutrient broth environment. The proportion of proteolytically-active cells in biofilms in the MUC5B environment was ten-times greater (47 ± 0.6%) than that for their planktonic counterparts (Figure 1b) whereas no such increase was seen in biofilms formed in the presence of nutrient broth. Imaging of the biofilms showed that *L. fermentum* cells adhered to both the uncoated and the MUC5B-coated surfaces and formed biofilms (Figure 2). In the MUC5B environment, proteolytically-active cells were evenly distributed throughout the biofilm (Figure 2a) whereas activity in the nutrient broth environment was low (Figure 2b). These data indicate that proteolytic activity is enhanced in biofilm cells of *L. fermentum* in a mucin-rich environment. The effect is not seen when planktonic *L. fermentum* cells are exposed to the same mucins in the fluid phase or in the presence of nutrient broth.

### Proteolytic activity in biofilm cells of *L. fermentum* is influenced by surface-associated MUC5B

**Figure 3:**
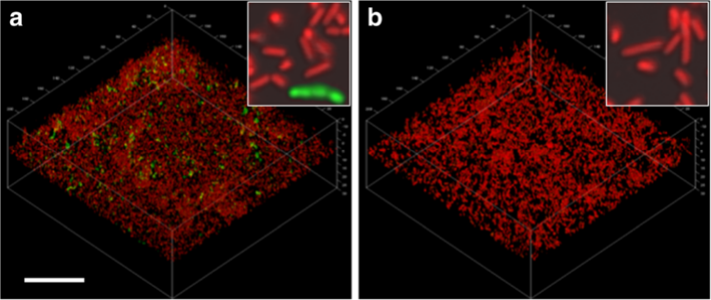
**Proteolytic activities of *Lactobacillus fermentum *biofilm populations in the presence of (a) surface-associated or (b) fluid-phase MUC5B mucins.** CSLM images showing biofilm cells growing in **(a)** the presence of a surface-coat of MUC5B mucins or **(b)** in the presence of MUC5B mucins in the fluid phase. Proteolytic activity was visualized by incubation with a FITC-conjugated casein substrate (green) for 1 hour. Cells were then counterstained using Syto 24 (red). The bar represents 20 μm and the inserts show four-fold enlargements of the main micrographs.

To determine whether the enhancement of proteolytic activity in the *L. fermentum* biofilm population in the MUC5B environment was mainly attributable to the surface-associated mucins or those in the bulk phase, the effects of these were investigated separately. Biofilms of *L. fermentum* were therefore either established on MUC5B-coated surfaces with nutrient broth in the fluid phase, or on uncoated surfaces with MUC5B in the fluid phase. In the presence of surface-associated MUC5B, image analysis revealed that 13 ± 3% of the population was proteolytically active (Figure 3) while in cells exposed to MUC5B in the fluid phase only, activity was very low (0.3 ± 0.02%). Thus, these data show that the presence of mucins on the surface was important for the enhancement of proteolytic activity. However, the proportion of active cells on the surface-associated mucins alone was significantly lower than that seen in the MUC5B environment, where mucins were also present in the bulk phase. This suggests that adherence to surface-associated MUC5B may have ‘primed’ the cells allowing them to respond to the presence of MUC5B in the fluid phase.

### Degradation of MUC5B by *L. fermentum*

**Figure 4:**
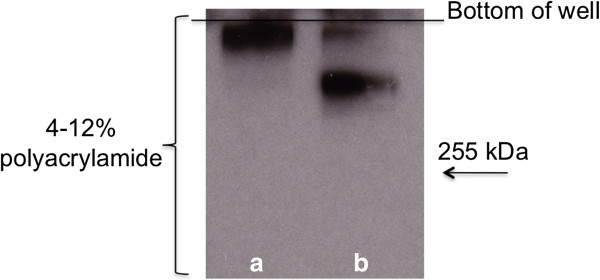
**Degradation of the MUC5B polypeptide backbone by proteases from biofilms of *L. fermentum.*** A control sample of MUC5B **(a)** and MUC5B which had been in contact with biofilm cells for 24 hours **(b)** were subjected to SDS-PAGE on 4–12% gels. After electro-blotting onto PVDF membranes, MUC5B was detected using the LUM5B-14 antiserum. The bottom of the well is indicated.

To determine whether mucins in the mucin-rich environment are degraded by the proteases produced within the *L. fermentum* biofilms, MUC5B mucins which had been in contact with biofilms for 24 hours were subjected to SDS-PAGE followed by Western blotting with an anti-MUC5B antiserum (Figure 4). Control mucins, which had not been in contact with bacteria, were visualized as a single band on top of the gel showing that they were of high-molecular-weight (>300 kDa). However when mucins had been incubated with *L. fermentum* biofilms, an additional band appeared in the upper region of the 4-12% gradient gel indicating that part of the mucins had undergone some degradation that allowed MUC5B to migrate into the gel. Thus these data confirm that proteolytically-active biofilm populations of *L. fermentum* can degrade MUC5B.

### Expression of surface proteins in *L. fermentum*

**Figure 5:**
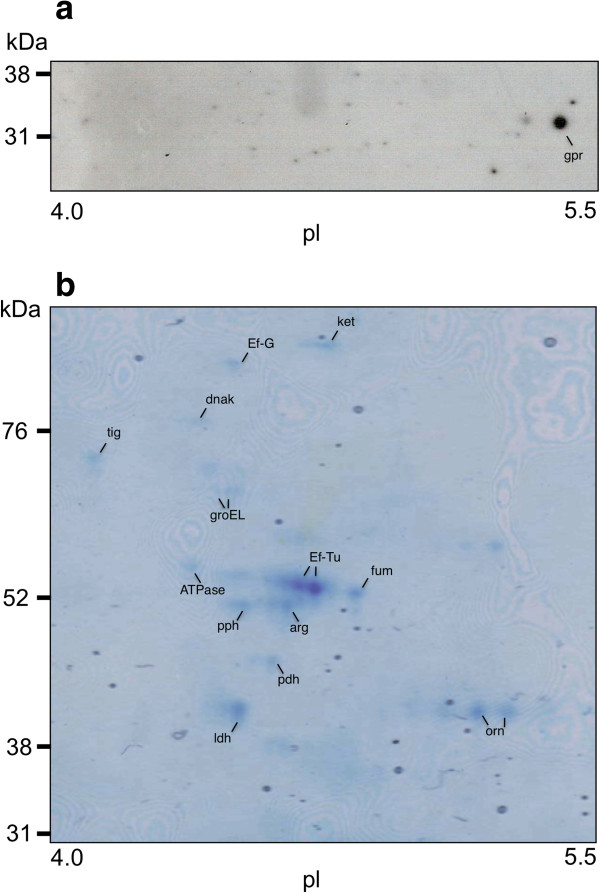
**Surface proteins identified in biofilm cells of *L. fermentum. *(a)** Western blotting of two-dimensional electrophoresis (2DE) of surface proteins. Samples were subjected to IEF in a pH 4–7 gradient followed by SDS-PAGE in 14% gels. After electroblotting onto PVDF membranes, *gpr,* O-sialoglycoprotein endopeptidase was detected using the polyclonal antiserum MOB-LFGE-2. **(b)** Gel stained with Coomassie Brilliant Blue after 2DE of surface proteins. Spots were excised from the stained gel, subjected to in-gel tryptic digestion and proteins identified by LC-MS/MS analysis. The proteins identified were: *arg*, arginine deiminase; *ATPase*, ATP synthase pyruvate dehydrogenase; *DnaK*; *Ef-G*, elongation factor G; *Ef-Tu*, elongation factor Tu; *fum*, fumarate hydratase; *GroEl*; *ldh*, lactate dehydrogenase; *ket*, phosphoketolase; *orn*, ornithine carbamoyltransferase; *pdh*, pyruvate dehydrogenase and *pph*, phophopyruvate hydratase.

**Figure 6:**
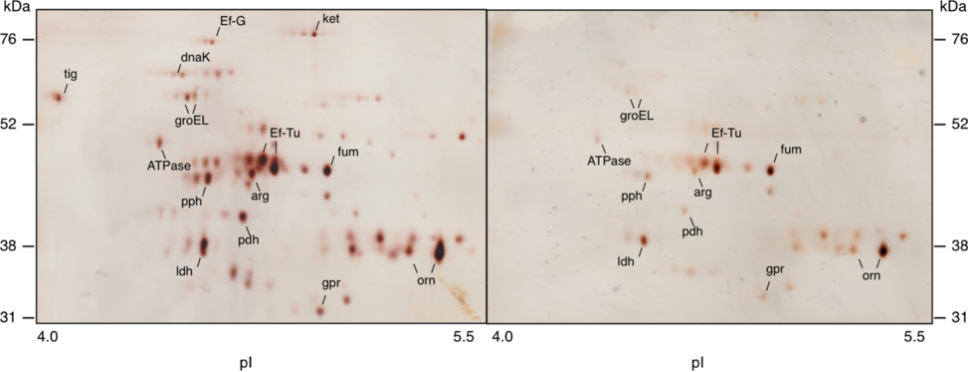
**Two-dimensional gel-electrophoresis of surface proteins from *Lactobacillus fermentum*.** Proteins isolated from bacteria in a MUC5B-environment (left) or a nutrient broth environment (right) were subjected to IEF in a pH 4–7 gradient followed by SDS-PAGE in 14% gels. Gels were silver-stained and image analysis of differently expressed proteins performed using Delta 2D software.

Since MUC5B mucins were degraded by biofilm cells of *L. fermentum*, the genome database was scanned for proteases capable of degrading glycoproteins. Using this approach, one protease, *O-*sialoglycoprotein endopeptidase, with a molecular weight of approximately 35 kDa, was selected as a possible candidate. Western blotting of 2DE gels of surface proteins from *L. fermentum* biofilm cells with a polyclonal antiserum raised against a peptide in the sequence of the glycopeptidase identified a spot at 32 kDa, corresponding to *O-*sialoglycoprotein endopeptidase (see Figure 5a). To investigate whether this protein was differentially expressed on the surface of *L. fermentum* biofilm cells in the different environments, surface proteins from biofilm cells in a MUC5B environment were compared with those from cells in a nutrient-broth-environment using 2D-gel electrophoresis. Image analysis of silver stained gels revealed that expression of the *O-*sialoglycoprotein endopeptidase was enhanced 2.7-fold in the presence of MUC5B, as compared to nutrient broth. In addition, a number of spots in the range 52–76 kDa showed higher levels of expression in the MUC5B- than in nutrient-broth-environment (see Figure 6 and Table 1). These were cut from a corresponding Coomassie-stained gel (see Figure 5b), subjected to LC-MS/MS and the proteins then identified by comparison of the tryptic fragments with the Mascot database. This revealed four proteins with a more than three-fold up-regulation in the MUC5B- as compared to the nutrient-broth-environments, corresponding to the chaperone proteins; trigger factor (25-fold increase), DnaK (9-fold increase), Ef-G (6-fold increase) and GroEL (3-fold increase). Thus these data show that besides up-regulation of the glycoprotease, increased proteolytic activity in the population is associated with an increase in the surface-expression of the chaperone proteins DnaK, GroEL and trigger factor.

**Table 1:** **Identification of the surface proteins in *L. fermentum *biofilms enhanced more than 2.0-fold in MUC5B environment compared to a nutrient broth environment**^**a**^

**Protein**	**IOD%**		**Fold change**^**b**^
	**MUC5B**	**Nutrient broth**	
Trigger factor	3.53	0.14	25.2
DnaK	2.57	0.29	8.9
Elongation factor G	2.91	0.46	6.3
GroEL	5.66	1.84	3.1
O-sialoglycoprotein endopeptidase	3.31	1.24	2.7
Pyruvate dehydrogenase	3.04	1.22	2.5

^a^Data obtained from analysis of the 2DE silver-stained gels shown in Figure 6.

^b^Fold change calculated as the percent value of the integrated optical density (IOD) of proteins in the MUC5B vs. the nutrient broth environment.

## Discussion

In their natural environments in the oral cavity, gastro-intestinal tract and female reproductive tracts, Lactobacilli are found in multi-species biofilms within the adherent mucus layers on the host mucosal surfaces. Survival and growth at these sites is dependent upon the ability of bacteria to bind to the mucus layers as well as to degrade complex substrates such as mucus glycoproteins to generate small peptides and saccharides as a source of nutrition. In this study, *L. fermentum* was able to bind and form biofilms in the MUC5B environment, in accordance with the expression of the mucin-binding protein, 32-Mmubp by this species [16]. The MUC5B mucin is a major component of adherent mucus layers in the oral cavity and the female reproductive tract, both sites at which *L. fermentum* is known to colonize [10,24].

The proportion of proteolytically-active cells within biofilm populations of *L. fermentum* was significantly higher in a mucin-rich environment than in the presence of nutrient broth suggesting that the presence of mucins promotes proteolytic activity in *L. fermentum*. The ability of MUC5B mucins to induce proteolytic activity has previously been demonstrated in the oral commensal bacterium, *Streptococcus mitis*[25] and up-regulation of proteolytic activity has also been shown in the fungal pathogen *Aspergillus fumigatus* growing in gastric mucins [26]. To determine the relative roles of surface-associated and fluid-phase mucins in mediating this enhanced activity, *L. fermentum* cells were allowed to adhere to surfaces coated with mucins or to form biofilms on non-mucin-coated surfaces where they were exposed to fluid-phase mucins alone. This revealed that exposure to fluid-phase mucins alone had no up-regulatory effect upon proteolytic activity in the biofilms. Likewise, proteolytic activity was not enhanced in planktonic cells exposed to mucins in solution, indicating that the presence of mucins in the fluid-phase does not up-regulate proteolytic activity in *L. fermentum*. In contrast, contact of the cells with surface-associated mucins caused a significant increase in proteolytic activity within the population suggesting that mucins play a pivotal role in regulating the effect and that the surface-associated molecules are of particular importance. The mechanisms underlying this observation are currently unknown but it is possible that epitopes within the mucins, revealed as a result of binding to a surface, are required for up-regulation of proteolytic activity. Interestingly, the level of proteolytic activity in biofilm cells on surface-associated mucins (13 ± 0.4%) was lower than that seen when mucins were present both on the surface and in the fluid-phase (47 ± 0.6%), suggesting that cells are able to further respond to the presence of fluid-phase molecules once they have been primed through contact with surface-associated ones.

Since the substrate used in this study is unlikely to penetrate the cells, the data presented here suggest that the proteolytic activity is the result of extracellular (surface-associated or secreted) proteases capable of degrading FITC-conjugated casein. Most of the available studies have focused on proteases in Lactobacillus species used in the production of fermented milk products. In these species, the best studied proteases are the cell-wall bound extracellular, caseinolytic proteases; PrtP, PrtB and PrtH (for a review see [17]) which degrade casein, into oligopeptides [27]. However, our analysis of the *L. fermentum* genome suggests that no homologue of these enzymes is present in this species. The proteases of *L. fermentum* have not been well described but the genome contains one good candidate for the proteolytic activity seen in this study, the glycoprotease - *O*-sialoglycoprotein endopeptidase. A polyclonal antiserum identified this protein in cell surface extracts from biofilm cells of *L. fermentum* and subsequent comparative proteomic analysis revealed that the glycoprotease was more highly expressed in the mucin environment than in the presence of nutrient broth. This enzyme has been described in detail from *Pasteurella haemolytica*, another commensal species from mucosal surfaces in the nasopharynx [28]. The glycoprotease, from *P. haemolytica* is a secreted metalloproteinase, which shows a high specificity for sialylated *O-*glycosylated glycoproteins, including P-selectins and glycophorin, but has also been shown to cleave casein. Interestingly, when sialic acid is removed, this enzyme is incapable of hydrolyzing glycoproteins [28-30], suggesting that the sialic acid residues are essential for the activity. The protein cores of the large gel-forming mucus glycoproteins such as MUC5B, contain regions with high levels of serine and threonine residues which carry *O-*linked glycans, many of which bear sialic acid residues [31]. The structure of MUC5B mucin would thus make it an ideal substrate for *O*-sialoglycoprotein endopeptidase. Up-regulation of protease activity in *L. fermentum* in the mucin environment was found to be associated with degradation of the MUC5B molecule, as confirmed using a mucin-specific antibody. This confirms that the protease produced by *L. fermentum* does degrade mucins and leads further weight to the argument that the enzyme responsible may be the *O*-sialoglycoprotein endopeptidase. *In vitro,* activation of this enzyme from *P. haemolytica* is facilitated by interaction with chaperone proteins such as DnaK which catalyse the appropriate folding events [32]. In this study, investigation of the cell-surface proteins associated with increased proteolytic activity revealed increased levels of the chaperone proteins DnaK, GroEL and trigger factor. Thus, export of chaperones to the cell surface may play a role in activation of the glycoprotease in response to MUC5B mucins.

## Conclusion

We have shown that a mucin-rich environment can enhance proteolytic activity in biofilms of *L. fermentum* and that this activity can cause mucin degradation. A possible mechanism for this effect is through increased synthesis and/or activation of *O-*sialoglycoprotein endopeptidase on the bacterial cell surface via interactions with chaperone proteins including DnaK. Activation of extracellular proteases in response to the presence of substrates such as mucins would provide one way for commensal lactobacilli such as *L. fermentum* to exploit complex substrates in their local environment in order to survive on mucosal surfaces.

## Abbreviations

CHAPS: 3-[(3-cholamidopropyl)dimethylammonio]-1-propanesulfonate; CSLM: Confocal scanning laser microscopy; 2DE: Two-dimensional SDS-polyacrylamide gel electrophoresis; DTT: Dithiethreitol; PBS: 0.15 M sodium chloride, 10 mM sodium dihydrogen phosphate, pH 7.4; PVDF: Polyvinylidene fluoride; TBST: Tris-buffered saline with Tween 20.

## Competing interests

The authors declare that they have no competing interests.

## Authors’ contributions

CW and LCdP participated in planning and designing the study, performed most of the laboratory work and participated in the data analysis and drafting of the manuscript. GS and JRD participated in study design, data analysis and drafting of the manuscript. All authors have read and approved the final manuscript.

## Pre-publication history

The pre-publication history for this paper can be accessed here:

http://www.biomedcentral.com/1472-6831/13/43/prepub
